# Anti-apoptotic Actions of Allopregnanolone and Ganaxolone Mediated Through Membrane Progesterone Receptors (PAQRs) in Neuronal Cells

**DOI:** 10.3389/fendo.2020.00417

**Published:** 2020-06-24

**Authors:** Peter Thomas, Yefei Pang

**Affiliations:** Marine Science Institute, University of Texas at Austin, Port Aransas, TX, United States

**Keywords:** membrane progesterone receptors, PAQR agonists, ganaxolone, allopregnanolone, inhibition apoptosis, neuronal cells, AED, neuroprotective

## Abstract

The neurosteroids progesterone and allopregnanolone regulate numerous neuroprotective functions in neural tissues including inhibition of epileptic seizures and cell death. Many of progesterone's actions are mediated through the nuclear progesterone receptor (PR), while allopregnanolone is widely considered to be devoid of hormonal activity and instead acts through modulation of GABA-_A_ receptor activity. However, allopregnanolone can also exert hormonal actions in neuronal cells through binding and activating membrane progesterone receptors (mPRs) belonging to the progestin and adipoQ receptor (PAQR) family. The distribution and functions of the five mPR subtypes (α, β, γ, δ, ε) in neural tissues are briefly reviewed. mPRδ has the highest binding affinity for allopregnanolone and is highly expressed throughout the human brain. Low concentrations (20 nM) of allopregnanolone act through mPRδ to stimulate G protein (G_s_)-dependent signaling pathways resulting in reduced cell death and apoptosis in mPRδ-transfected cells. The 3-methylated synthetic analog of allopregnanolone, ganaxolone, is currently undergoing clinical trials as a promising GABA-_A_ receptor-selective antiepileptic drug (AED). New data show that low concentrations (20 nM) of ganaxolone also activate mPRδ signaling and exert anti-apoptotic actions through this receptor. Preliminary evidence suggests that ganaxolone can also exert neuroprotective effects by activating inhibitory G protein (G_i_)-dependent signaling through mPRα and/or mPRβ in neuronal cells. The results indicate that mPRs are likely intermediaries in multiple actions of natural and synthetic neurosteroids in the brain. Potential off-target effects of ganaxolone through activation of mPRs in patients receiving long-term treatment for epilepsy and other disorders should be considered and warrant further investigation.

## Introduction

**Figure 1:**
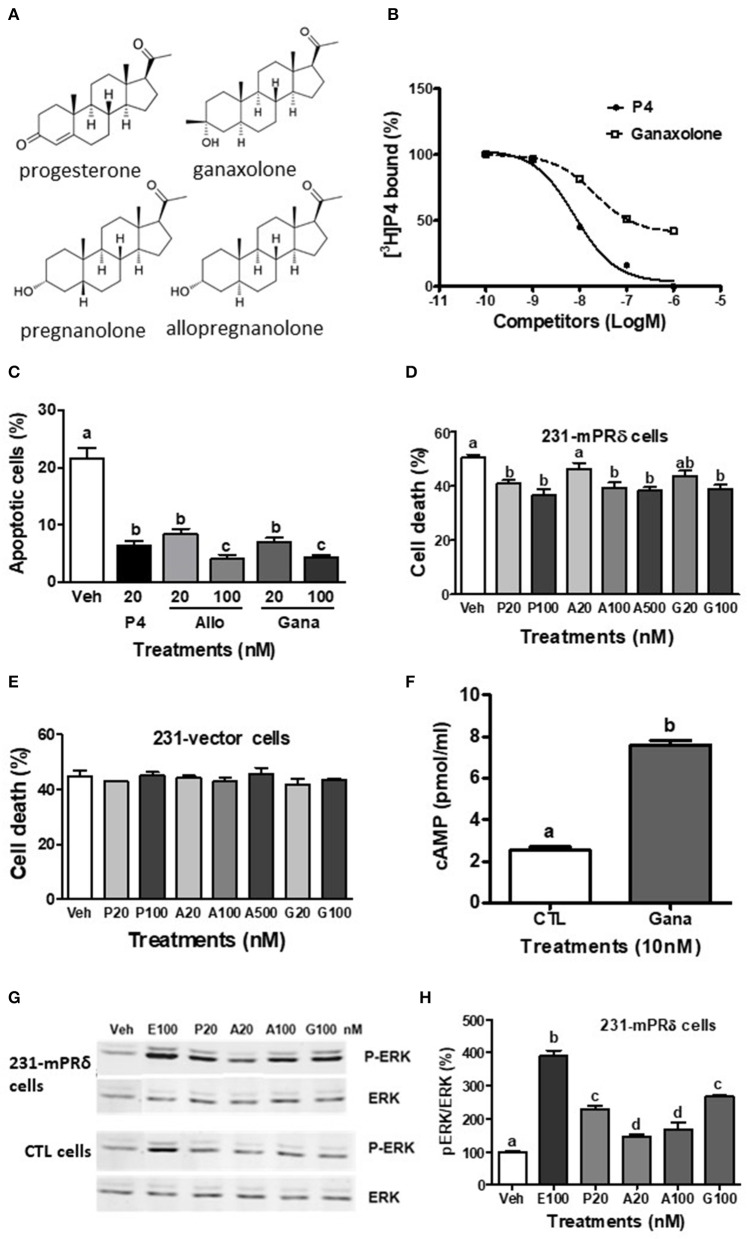
Interactions of ganaxolone with human mPRδ (PAQR6). **(A)** Structures of ganaxolone and several other neurosteroids. **(B)** Representative competition curve of ganaxolone binding to plasma membranes of mPRδ-transfected MDA-MB-231 cells (231-mPRδ) expressed as percentage of maximum [^3^H]-progesterone binding. Ganaxolone was added to the assay buffer dissolved in ethanol. Ethanol was 0.1% of total volume, which did not affect [^3^H]-progesterone binding. P4, progesterone. **(C,E,F)** Effects of 4 days treatment with progesterone (P4), allopregnanolone (Allo) and ganaxolone (Gana) on serum starvation-induced percent apoptotic cells detected with a TUNEL assay kit **(C)** and percent cell death detected by trypan blue staining **(E,F)** of the vector-transfected MDA MB-231 cells and 231-mPRδ cells, *N* = 3. **(D)** Effect of treatment with ganaxolone (10 nM) for 15 min. on cellular cAMP levels in 231-mPRδ cells. *N* = 3. **(G,H)** Representative Western blot analysis and quantification of effects of 20-min treatments with progesterone (P), allopregnanolone (A), and ganaxolone (G) on activation of ERK. P-ERK: phosphorylated ERK, ERK: total ERK in the vector- (CTL cells), and 231-mPRδ cells. E100: 100 nM EGF as a positive control. The bar graph shows relative densitometry changes of the bands in Western blot images (*N* = 3). Results were analyzed by one-way ANOVA, followed by Newman-Keul's multiple comparison test. Treatment groups that are significantly different from each other in the *post hoc* test (*P* < 0.05) are indicated by different letters. Experiments were repeated three or more times, and similar results and similar significant differences between treatment groups were obtained on each occasion. See Pang et al. (15) for descriptions of reagents, culture conditions and assay procedures.

Progesterone and its metabolites produced in neural tissues (neurosteroids, Figure 1A) such as allopregnanolone mediate a wide variety of actions in the brain including neuroprotection, anti-apoptosis, inhibition of epileptic seizures, reproductive behaviors, neuroendocrine control of reproduction, and both pro-tumorigenesis and anti-tumorigenesis (1–3). Many genomic actions of progesterone in neural tissues are mediated through PR whereas the neurosteroid allopregnanolone has negligible binding affinity for the PR and instead interacts with GABA-_A_ receptors resulting in decreases in their activities and also activates the pregnane X receptor (PXR) (3–6). However, progesterone actions have also been observed in the brain which are PR-independent (i.e., persist in PR knockout mice) and in neuronal cells which have low expression of PRs (e.g., GnRH-producing GT1-7 cells) (7–9). Evidence has accumulated that some of these actions may be mediated through membrane progesterone receptors (mPRs) (4, 10, 11), 7-transmembrane receptors coupled to G proteins belonging to the progestin and adipoQ receptor (PAQR) family which is unrelated to the GPCR superfamily (12, 13). Moreover, recent studies with cultured neuronal cells show that low concentrations of progesterone and allopregnanolone exert hormonal actions through binding and activating mPRs, resulting in rapid induction of intracellular signaling pathways and anti-apoptosis (14, 15). Collectively, these results suggest that mPRs are likely intermediaries of progesterone and allopregnanolone actions in neural tissues, with potential implications for human health and disease.

The mechanisms by which progesterone, allopregnanolone, and an antiepileptic drug, ganaxolone, exert their protective actions in epilepsy are summarized here. The characteristics of mPRs, their distribution in brains of humans and rodents, and their proposed functions in the central nervous system are briefly discussed. The anti-apoptotic actions of allopregnanolone in neuronal cells and in mPR-transfected cancer cells that are mediated through mPR-dependent signaling pathways are reviewed. Ganaxolone, a synthetic analog of allopregnanolone, is currently undergoing clinical trials as a third generation AED that targets GABA-_A_ receptors (16, 17). New data are presented showing that ganaxolone binds to mPRs and mimics the anti-apoptotic actions of allopregnanolone and progesterone in these cultured cells. These results indicate that clinical studies with ganaxolone should include investigations of possible additional unexpected, off-target effects of the drug through activation of mPRs.

## Protective Effects of Neurosteroids Against Epileptic Seizures

Epilepsy is a severe neurological disorder that affects over 50 million people throughout the world (16, 18). Progesterone exerts anticonvulsant effects in animal epilepsy models through a PR-independent mechanism as they are not decreased in PR knockout (PRKO) mice (19). Instead, progesterone's anticonvulsant potency is increased in PRKO mice which is consistent with results showing activation of PR in a status epilepticus rat model increases seizure frequency (19, 20). Progesterone's antiseizure actions are dependent on its conversion to allopregnanolone since cotreatment with the 5α-reductase inhibitor, finasteride, blocks progesterone's actions (19). Allopregnanolone displays very weak binding affinity for PRs (21) and exerts its protective effects against seizures through a PR-independent mechanism. Allopregnanolone acts through positive allosteric modulation of GABA-_A_ receptor activity, and can also activate the receptors at higher concentrations in the absence of GABA (22, 23). Positive modulation of GABA-_A_ receptors by allopregnanolone enhances inhibitory chloride conductance which in turn decreases neuronal excitability and reduces the incidence of seizures (19, 24). Although over 20 AEDs have been used to treat this disease, these treatments are ineffective in controlling seizures in a third of epileptic patients and long-term treatment with enzyme-modulating AEDs can cause endocrine, metabolic, and reproductive disorders (25). The 3β-methylated synthetic analog of allopregnanolone, ganaxolone (3α-hydroxy-3β-methyl-5α-pregnan-20-one, Figure 1A) is a promising fourth generation AED that is currently completing phase III clinical trials (16). Ganaxolone has been shown to have activity in several animal epilepsy models and is effective in infants with spasm and in adults with partial-onset seizures (17). Ganaxolone is also potentially useful for treatment of mood and anxiety disorders (16) and has been shown to improve sociability in a rodent model of autism spectrum disorder, which indicates it may increase sociability in autistic patients (26). Although ganaxolone can cause sedation in epilepsy patients, few other adverse effects of long-term administration of the drug have been observed to date in clinical trials. Methylation at the 3β position of ganaxolone impairs its metabolism to inactive metabolites, thereby increasing its period of effectiveness in inhibiting seizures compared to allopregnanolone (26). Like allopregnanolone, ganaxolone is an allosteric modulator of GABA-_A_ receptors and acts through different allosteric binding sites to that of benzodiazepines, as revealed by ligand binding assays and receptor mutational analysis (17, 27, 28).

## Membrane Progesterone Receptors (mPRs, PAQRs)

Progesterone exerts hormonal actions in numerous cell and animal models through activation of membrane progesterone receptors (mPRs) belonging to the progesterone and adipoQ receptor (PAQR) family (29). These novel 7-transmembrane receptors were initially discovered in teleost fish gonads and their homologs were subsequently identified in other vertebrate classes (30, 31). mPRs mediate rapid, non-classical progesterone actions, which are frequently non-genomic, by activating G proteins and modulation of intracellular signaling pathways. The five mPR members of the PAQR family, mPRα (PAQR7), mPRβ (PAQR8), mPRγ (PAQR5), mPRδ (PAQR6), and mPRε (PAQR9), have different tissue distributions, progestin binding specificities, signal transduction pathways, and functions in vertebrate cells and tissues (12–15). mPRα is the predominant mPR isoform expressed in most progesterone target tissues with the exception of the brain and is the primary mPR that regulates several critical reproductive and non-reproductive progestin functions. For example, mPRα mediates oocyte meiotic maturation and sperm motility in fish, anti-apoptosis in fish ovarian granulosa cells and in human breast cancer cells (32, 33), relaxation of human myometrial and vascular muscle cells (34, 35), reversal of epithelial to mesenchymal transition in breast cancer cells (36), and inhibition of prolactin release from rat lactotrophs through activation of TGFβ1 (37).

## Localization of mPRs in the Brain and Peripheral Nervous System

All five mPRs subtypes are expressed throughout the human brain and relative expression of mPRδ mRNA is highest among all the mPRs in nearly all brain regions, with greatest expression in the corpus callosum, hypothalamus, and spinal cord. Furthermore, mPRδ mRNA expression is greater than the mRNA expression of the other mPRs in the neocortex lobes, the limbic system (amygdala, hippocampus, nucleus accumbens), thalamus, as well as in the caudate and putamen, substantia nigra, medulla, and pons, brain regions involved in memory and movement, reward, and autonomic functions (15). Expression of mPRβ and mPRε genes is also high in many of these regions, including the hypothalamus, hippocampus, caudate, cerebellum, pons, and spinal cord, whereas mPRα expression is lower in most brain regions with highest expression in the temporal lobe, medulla, and spinal cord and mPRγ expression is low throughout the brain with the exception of the pons, spinal cord and choroid plexus. The finding that PR mRNA is also expressed throughout the human brain indicates the potential for interactions between mPRs and PR in progesterone regulation of brain functions. However, PR mRNA expression is lower than that of the mPRs in all brain regions except in the pituitary gland which also expresses high levels of mPRε (15).

Unfortunately, the only information currently available on mPRδ and mPRε expression in rodent brains is in the mouse hypothalamus, where low mRNA levels of these subtypes and mPRγ were detected, <10% those of mPRα and mPRβ (38). However, mPRα and mPRβ are broadly distributed in rat and mouse brains (4), with mPRα expression detected in the hippocampus, cerebellum, hypothalamus, thalamus, cortex, striatum, and olfactory bulb (39). Higher mRNA expression of mPRβ compared to that of mPRα and high immunoreactive mPRβ protein expression have been reported in the cortex, paraventricular, and preoptic regions of the hypothalamus, the oculomotor nucleus in the mesencephalon, with substantial expression also in the telencephalon, hippocampus, thalamus, and pons of female rat brains (40, 41). Although these two mPR subtypes are expressed only in neurons under normal conditions, mPRα is also expressed in oligodendrocytes, astrocytes and glial cells after traumatic brain injury, suggesting a potential role for the receptor in inflammatory responses and myelin repair (39). Similarly, mPRα is expressed in astrocytes, oligodendrocytes and their progenitor cells as well as in neurons throughout the spinal cord, whereas mPRβ has a more limited distribution and is mainly located in ventral horn neurons and neurites (42). Interestingly, in the peripheral nervous system all five mPR isoforms have been detected in Schwann cells (43). Collectively, these results indicate that progesterone and possibly allopregnanolone can act in all human brain regions through mPRs and suggest that different mPR subtypes are major intermediaries in these neurosteroid actions within distinct brain regions.

## Functions of mPRs in the Brain and Peripheral Nervous System

Although relatively few studies have been conducted so far on mPR functions in the brain, there is emerging evidence that they are intermediaries in several important progesterone neural functions. Experiments in new-born rats with the mPR-selective agonist, Org OD 02-0 (02-0) and in adults injected with mPRβ si-RNA show that the receptor is involved in stabilizing breathing and reducing apnea (9, 44, 45). Knockdown of mPRβ and mPRα mRNAs in the midbrains of female adult rodents by injection of antisense oligonucleotides into the lateral ventricle decreased reproductive behaviors (lordosis and aggression/rejection), whereas other behaviors were not affected (10, 46). The mPR agonist, 02-0, increases dopamine release from hypothalamic explants of rodent prolactinoma models resulting in decreased prolactin secretion and also exerts a direct action on mPRα in pituitary lactotrophs to decrease prolactin secretion through TGFβ-1 (37, 38). These results suggest mPR agonists are potentially of therapeutic use for treating pathological hyperprolactinemia (47). However, the role of mPRs in tumorigenesis in the brain remains unclear. Whereas, proliferation and invasion of glioblastoma cells was stimulated by 02-0 and decreased when mPRα expression was silenced (48), progesterone has been shown to inhibit the growth and metastasis of PR-null breast cancer cells through mPRα in the brains of nude mice (49). Several mPR functions have also been identified in the peripheral nervous system. A recent study showed migration and proliferation of primary rat Schwann cells *in vitro* were increased by treatment with the mPR agonist, 02-0 (50). This treatment also increased expression of differentiation markers and caused morphological changes characteristic of the repair phenotype, indicating a potential role of mPRs in peripheral nerve regeneration following injury (50). Activation of mPRs has also been shown to promote neurite growth in PC12 cells (51).

## Interactions of Allopregnanolone With mPRs

We have shown that allopregnanolone exerts protective effects through mPR-dependent signaling pathways in cultured breast cancer cells that do not express PR or GABA-_A_ receptors as well as in neuronal cells (14, 15). Among the mPRs allopregnanolone displays the highest binding affinity for mPRδ in transfected MDA-MB-231 triple negative breast cancer cells (231-mPRδ) with an IC50 of 151 nM and a relative binding affinity (RBA) of 33.6% that of progesterone. Allopregnanolone is also an effective competitor for [^3^H]-progesterone binding to mPRα and mPRβ, with IC50s of 400 and 550 nM, respectively, whereas it has negligible binding affinity for mPRε. Another neurosteroid, 5α-dihydroprogesterone, also displays high binding affinity for mPRα (13). Interestingly, the neurosteroids, dehydroepiandrosterone and pregnanolone (52) (Figure 1A), have relatively high binding affinities for mPRδ, with IC50s of 780 and 346 nM, respectively (15). A low concentration of allopregnanolone (20 nM) was shown to mimic the stimulatory actions of progesterone on cAMP production and ERK phosphorylation and also attenuate serum starvation-induced cell death and apoptosis in 231-mPRδ cells cultured *in vitro*. Allopregnanolone (20 nM) also decreased apoptosis of rat hippocampal neuronal (H19-7) cells which express mPRδ and mPRε, whereas the PR agonist R5020 was ineffective. Interestingly, allopregnanolone also mimicked the actions of progesterone in cultured rat GnRH secreting GT1-7 cells which express high levels of mPRα and mPRβ and lower expression of mPRδ mRNA to decrease cAMP production and attenuate cell death and apoptosis (14). The finding that allopregnanolone causes a decrease in cAMP production in GT1-7 cells suggests it is acting through mPRα and/or mPRβ which activate a Gi in these cells, rather than through mPRδ which activates a stimulatory G protein (7, 15). Collectively, these results suggest that low physiological concentrations of allopregnanolone can also potentially act through mPRα/mPRβ to influence their neural functions. On the basis of these findings we hypothesized that ganaxolone can similarly alter neuronal cell functions mediated by mPRs. Therefore, in the present study we investigated whether ganaxolone binds to mPRs, activates mPR-dependent signaling, and exerts anti-apoptotic actions in several neuronal cell lines and in mPR-transfected cells. Experiments were conducted primarily with 231-mPRδ cells, since mPRδ displays the highest binding affinity for allopregnanolone, following experimental procedures described in detail previously (15).

## Interactions of Ganaxolone With mPRs

**Figure 2:**
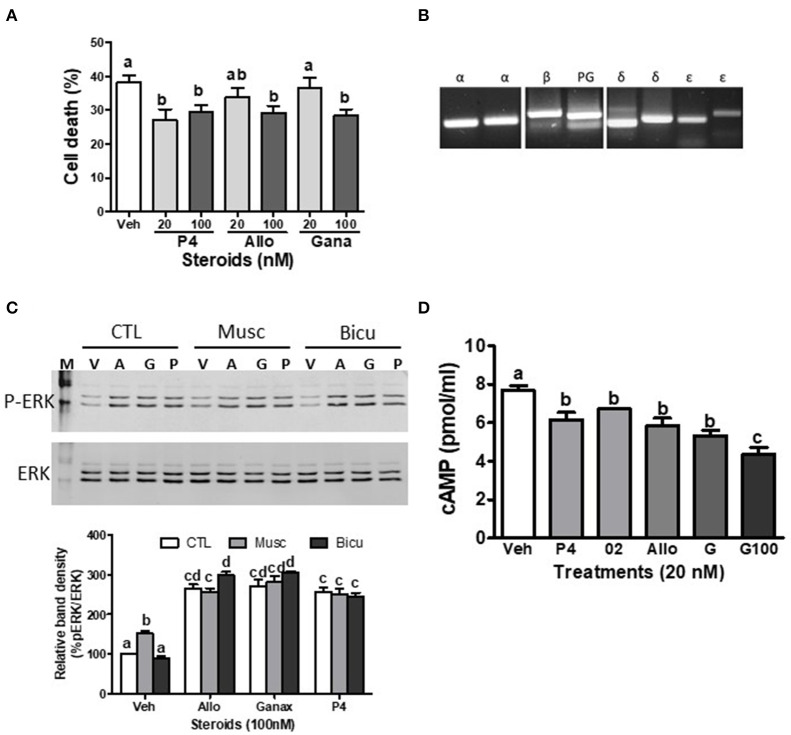
Effects of ganaxolone on rodent neuronal cell lines. **(A)** Effects of 4 days treatment with 20 nM and 100 nM progesterone (P4), allopregnanolone (Allo) and ganaxolone (Gana) on cell death of mouse hypothalamic GT1-7 cells. *N* = 3. **(B)** Detection of mPRα (α), mPRβ (β), mPRδ (δ), mPRε (ε), and progesterone receptor membrane component 1, PGRMC1 (PG) mRNA expression by RT-PCR in immortalized rat hippocampal H19-7 cells. **(C)** Representative Western blot analysis of effects of pre-incubation with muscimol (Musc, 100 μM) and bicuculline (Bicu, 1 μM) for 20 min on neurosteroid-induced (100 nM, for 20 min.) activation of ERK. P-ERK, phosphorylated ERK; ERK, total ERK in H19-7 cells; V, vehicle control; A, allopregnanolone; G, ganaxolone; P, progesterone. The bar graph shows relative densitometry changes of the bands in Western blot images (*N* = 3). **(D)** Effects of 15 min. treatments with 20 nM progesterone (P4), Org OD 02-0 (02), allopregnanolone (Allo) and ganaxolone (G, 20 and 100 nM) on cAMP levels in H19-7 cells. (*N* = 3). Results were analyzed by one-way ANOVA, followed by Newman-Keul's multiple comparison test. Treatment groups that are significantly different from each other in the *post hoc* test (*P* < 0.05) are indicated by different letters. Experiments were repeated three or more times, and similar results and similar significant differences between treatment groups were obtained on each occasion. See Pang et al. (15) for descriptions of reagents, culture conditions, and assay procedures.

The present results show that ganaxolone also binds to mPRs and displays agonist activity in 231-mPRδ cells that do not express GABA-_A_ receptors (Figure 1). A representative competitive binding assay showed that ganaxolone displaced up to 60% of [^3^H]-progesterone binding to cell membranes of 231-mPRδ cells (Figure 1B) with an approximate IC50 of 100 nM, similar to that for allopregnanolone (15). However, the ganaxolone competition curve was not parallel to that of progesterone and higher ganaxolone concentrations (10^−7^ and 10^−6^M) were ineffective in displacing the remaining ~40% of [^3^H]-progesterone binding. Similarly, a previous study showed that higher concentrations of allopregnanolone (10^−6^ and 10^−5^ M) did not replace the residual 30% [^3^H]-progesterone binding to mPRδ (15). One possible interpretation of the results is that these two neurosteroids do not occupy all the progesterone binding sites on mPRδ. However, additional research on their potential binding to allosteric sites as well as their interactions with progesterone binding to orthosteric sites will be required to determine the nature of their interactions with mPRδ, and whether, for example, they act as ago-allosteric ligands (28, 53, 54). The results indicate that ganaxolone, like allopregnanolone, can potentially influence progesterone signaling through mPRδ. The finding that a low concentration of ganaxolone (20 nM) mimicked the effects of 20 nM progesterone and allopregnanolone on inhibition of serum starvation-induced apoptosis (Figure 1C) and cell death in 231-mPRδ cells (Figure 1E), whereas it was ineffective in reducing cell death in vector-transfected 231 cells (231-vector, Figure 1F) demonstrates that ganaxolone has a mPRδ agonist activity at low nM concentrations. While the progesterone-induced decreases in the two assays were similar (10–12% of the cells), percent cell death measured by trypan blue exclusion was higher than the percent apoptotic cells, which was expected because this assay does not distinguish between cell mortality and cell morbidity after serum starvation, whereas the TUNEL assay is a more robust measure of cells undergoing programed cell death (33). Ganaxolone triggers the same intracellular signaling pathways as those activated by progesterone and allopregnanolone. Ganaxolone treatment (10 nM) increased cAMP levels more than two-fold over no treatment control values in 231-mPRδ cells, consistent with previous results showing mPRδ activates a stimulatory G protein (Figure 1D) (15). Ganaxolone (100 nM) ganaxolone mimicked the effects of progesterone and allopregnanolone on phosphorylation of ERK in 231-mPRδ cells (Figures 1G,H). At the higher concentration (100 nM), ganaxolone also mimicked the inhibitory effects of progesterone and allopregnanolone on serum starvation-induced cell death in rat hypothalamic GT1-7 cells (Figure 2A). GT1-7 cells do not express appreciable amounts of PR mRNA in the absence of estrogen priming, but display significant expression of mPRα and mPRβ which have relatively high binding affinities for allopregnanolone, and lower expression of mPRδ (7, 14, 15). Rat hippocampal neuronal H19-7 cells, in which progesterone and allopregnanolone have previously been shown to inhibit serum starvation-induced cell death (15), have low expression of the PR and high expression of mPRα, mPRβ and mPRδ (Figure 2B). All three neurosteroids caused significant phosphorylation of ERK in H19-7 cells (Figure 2C). Moreover, MAP kinase signaling and its activation by ganaxolone and the two other neurosteroids was not altered by pretreatment with 100 μM muscimol, a GABA-_A_ receptor agonist, or with 1 μM bicuculline, a GABA-_A_ receptor antagonist (Figure 2C), confirming that activation of this pathway by these neurosteroids in H19-7 cells is not mediated through a GABA-_A_ receptor. Moreover, the finding that the PR agonist R5020 does not have the anti-apoptotic effects observed with progesterone and allopregnanolone in H19-7 cells (15), suggests the PR is not involved in this response. Interestingly, treatments with 20 nM ganaxolone and allopregnanolone mimicked the effects of progesterone and the mPR-selective agonist, 02-0, to decrease cAMP production in H19-7 cells (Figure 2D), indicating an inhibitory G protein is activated. These results suggest these neurosteroids act through mPRα and/or mPRβ in H19-7 cells, rather than through mPRδ, since mPRα and mPRβ activate inhibitory G proteins.

## Discussion

There is an extensive body of literature describing neuroprotective functions of progesterone and allopregnanolone mediated through the PR and GABA-_A_ receptors, respectively. Our results suggest that allopregnanolone and the synthetic neurosteroid, ganaxolone, can also exert protective functions in cultured neuronal cells through activation of mPRs to attenuate cell death and apoptosis. However, details of the signaling pathways activated by these steroids through mPRs in neuronal cells are lacking. Moreover, only limited information is currently available on the functions of mPRs in the central nervous system and there is an urgent need to determine whether these neurosteroids exert similar neuroprotective functions through mPRs in *in vivo* models of neurodegenerative diseases. Information is also lacking on possible interactions between mPR and other progesterone and allopregnanolone signaling pathways mediating neuroprotective functions in neural tissues. For example, progesterone membrane component 1, which is abundant in many brain regions (4) and has been proposed to mediate progesterone neuroprotective actions (55), acts as an adaptor protein for mPRα in breast cancer cells, by coupling to mPRα and facilitating its transport to the cell surface where it mediates its membrane receptor functions (56). In addition, cross-talk between mPR and PR signaling has been shown in human myometrial cells and in rat Schwann cells. Activation of mPR in myometrial cells causes transactivation of PR and modulation of PR coactivator expression (34). On the other hand, experiments with the PR agonist, R5020, show mPRα and mPRβ expression in rat primary Schwann cells can be upregulated through the PR (50). Finally, it is important to obtain a clearer understanding of possible off-target effects of ganaxolone through activation of mPRs which would indicate several additional physiological functions that should be monitored in future clinical trials as well as suggesting potential medical complications for some epilepsy patients after long-term ganaxolone treatment.

## Data Availability Statement

The raw data supporting the conclusions of this article will be made available by the authors, without undue reservation.

## Author Contributions

The manuscript was written by PT and edited by YP. This study was designed by PT and the experiments were conducted and analyzed by YP. The interpretation of the results was conducted by PT and YP. All authors contributed to the article and approved the submitted version.

## Conflict of Interest

The authors declare that the research was conducted in the absence of any commercial or financial relationships that could be construed as a potential conflict of interest.
